# Primary lung tumour visualised by transthoracic echocardiography

**DOI:** 10.1186/1476-7120-6-60

**Published:** 2008-12-16

**Authors:** Magnus Dencker, Carin Cronberg, Sabine Damm, Sven Valind, Monica Wadbo

**Affiliations:** 1 Department of Clinical Physiology and Nuclear Medicine, Malmö University Hospital, Lund University, Malmö, Sweden; 2 Department of Radiology, Malmö University Hospital, Lund University, Malmö, Sweden; 3 Department of Respiratory Medicine and Allergology, Malmö University Hospital, Lund University, Malmö, Sweden

## Abstract

We present images of a rare case where a primary lung tumour was visualised by transthoracic echocardiography. The patient was a 78-year-old male where Chest X-ray had revealed a tumour-suspected structure in the left lung. Both transthoracic echocardiography and combined PET/CT images showed a large tumour located close to the heart. Fine-needle biopsy showed non-small cell lung cancer.

## Introduction

Echocardiography has a vital role for monitoring cardiac function. The field of view is, however, limited when it comes to a structure that has extracardiac location. It is well known that extracardiac structures such as hiatal hernias, pleural effusions or ascites can be evaluated with transthoracic echocardiography [1]. We present images of a rare case where a primary lung tumour was visualised by transthoracic echocardiography.

## Case presentation

**Figure 1:**
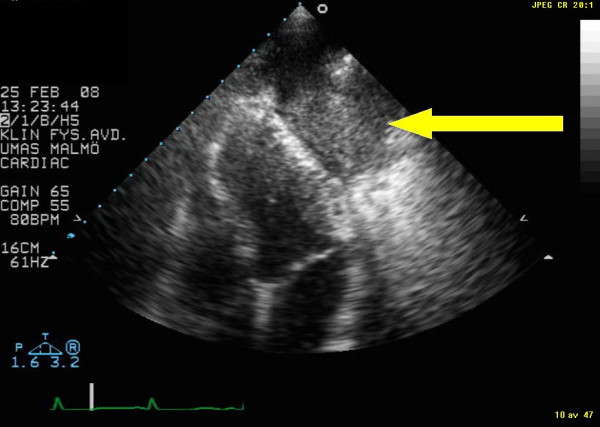
Display of echocardiography image from apical four-chamber view. The arrow indicates the non-small cell lung cancer.

**Figure 2:**
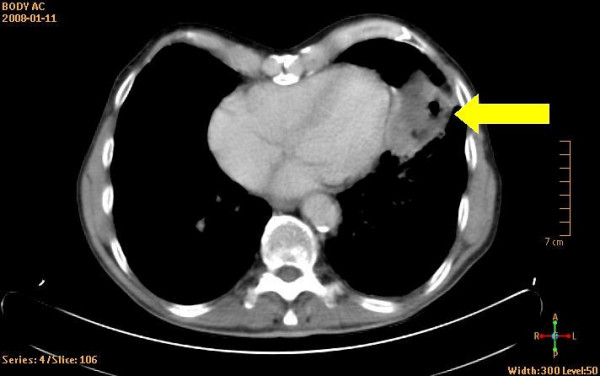
Display of CT-image. The arrow indicates the non-small cell lung cancer.

**Figure 3:**
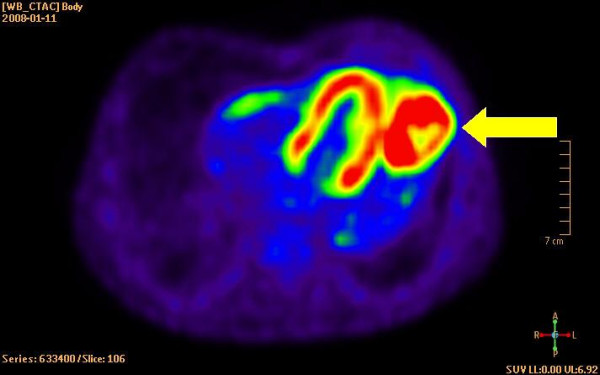
Display of PET-image. The arrow indicates the non-small cell lung cancer.

The patient was a 78-year-old male with a history of smoking up until seven years ago. He was under treatment for hypertension and rheumatoid arthritis. Chest X-ray had revealed a tumour-suspected structure in the left lung. Standard transthoracic echocardiography examination was performed, as part of a cardiac evaluation, with Sonos 5500 (Philips Medical Systems, Best, The Netherlands). The patient had poor acoustic windows. A predominantly echolucent structure was visualised from apical four-chamber view (Figure 1 and Additional file 1). The structure measured approximately 4 × 7 centimetres and was located apical/laterally to the left ventricle. For primary tumour staging the patient underwent a combined computed positron emission tomography technique (PET) and tomography (CT) examination by an integrated PET/CT system (Gemini TF, Philips Medical Systems, Best, The Netherlands) after injection of 276 MBq of ^18^F-fluorodeoxyglucose (FDG). The use of a radiopharmaceutical such as FDG provides the capability for imaging tumour glucose metabolism, whereas CT images give anatomical information [2]. Figure 2 displays a CT image that shows the rather large tumour located close to the heart. Figure 3 is the corresponding PET image that displays FDG uptake. Fine-needle biopsy showed a non-small cell lung cancer.

## Discussion

The only reason that the primary lung tumour was visualised by transthoracic echocardiography was due to the close proximity to the heart and therefore in the field of view. It would be rather preposterous to suggest that transthoracic echocardiography could be a method for visualization of primary lung tumour. This case highlights, however, the importance of careful inspection of extracardiac space when performing transthoracic echocardiography. Most case reports of echocardiography and lung tumours are about various presentations of metastases, which may not be surprising since primary lung cancer is one of the most prevalent sources of cardiac metastases. Previous reports include lung cancer metastases that led to ECG alterations mimicking acute lateral or antero-lateral ST-elevation infarction [3,4]. Also, invasive growth of lung cancer to the left atria through pulmonary vein has been described [5]. Another echocardiography presentation of intra thoracic tumours has been compression pulmonary artery or pulmonary vein [6,7]. Combined PET/CT images are becoming the method of choice for staging of primary non-small cell cancer [2]. Use of a radiopharmaceutical such as FDG provides the information of tumour metabolism, whereas CT images give anatomical information. The principal use has been for primary staging and because of the high negative predictive value reducing the number of mediastinoscopys.

## Consent

Written informed consent was obtained from the patient for publication of this case report.

## Competing interests

The authors declare that they have no competing interests.

## Authors' contributions

MD performed the echocardiography and wrote the manuscript. CC, SD, and SV were responsible for the PET/CT. MW was the treating physician. All authors approved the final version of the manuscript.

## Supplementary Material

Additional file 1 Transthoracic echocardiography from apical four-chamber view. Note the structure located apical/laterally to the left ventricle, measured approximately 4 × 7 centimetres and represented the non-small cell lung cancer. Click here for file
